# Optimizing Temperature in Ex Situ Heart Perfusion: A Comparative Review of Traditional and Novel Methods in Heart Transplantation

**DOI:** 10.3390/jcdd13010025

**Published:** 2026-01-01

**Authors:** Panos Georghiou, Georgios P. Georghiou, Cristiano Amarelli, Marius Berman

**Affiliations:** 1 Barts and The London School of Medicine and Dentistry, Queen Mary University of London, Turner St., London E1 2AD, UK; panos.georghiou@gmail.com; 2 Department of Cardiothoracic Surgery, School of Medicine, European University Cyprus, 6 Diogenous St., Egkomi, 2404 Nicosia, Cyprus; geo.georgiou@euc.ac.cy; 3 Department of Cardiac Surgery and Transplants, AORN dei Colli Monaldi Hospital, Via Leonardo Bianchi, 80131 Naples, Italy; cristiano.amarelli@ospedalideicolli.it; 4 Department of Transplantation, Royal Papworth Hospital, Papworth Rd, Cambridge CB2 0AY, UK

**Keywords:** heart transplantation, heart failure, ex situ heart perfusion, machine perfusion (MP), normothermic perfusion (NMP), hypothermic perfusion (HMP), subnormothermic perfusion (SNMP), static cold storage (SCS), primary graft dysfunction (PGD), ischemia–reperfusion injury (IRI)

## Abstract

Heart transplantation is still the definitive therapy for end-stage heart failure, yet the persistent shortage of suitable donor organs limits its application. Traditionally, static cold storage (SCS) has served as an effective standard preservation method, providing safe and adequate protection for preservation times under four hours. Yet, the need to extend this window and the specific metabolic requirements of donation after circulatory death (DCD) hearts have prompted interest in machine perfusion (MP) technologies. This literature review investigates the influence of temperature in ex situ heart perfusion, comparing normothermic (NMP), hypothermic (HMP), and subnormothermic (SNMP) strategies. Evidence from experimental models and emerging clinical studies suggests that MP can prolong preservation times, mitigate ischemic injury, and enable real-time metabolic and viability assessment of donor hearts prior to transplantation. These strategies represent a central trade-off: NMP enables real-time functional assessment of the beating heart, while HMP and SNMP approaches prioritize profound metabolic suppression to mitigate ischemic injury. Nonetheless, current data are limited by high costs, significant resource requirements, variability in perfusion protocols, and the scarcity of randomized controlled trials, particularly for HMP and SNMP. Standardization of methodologies, direct comparative studies, and the adoption of a risk-stratified preservation ecosystem are needed to clarify optimal temperature strategies. However, recent clinical successes with hypothermic strategies in traditionally normothermia-dependent donor types, such as DCD hearts, signal a potential paradigm shift, challenging established value propositions and prompting a critical re-evaluation of optimal preservation strategies.

## 1. Introduction

Heart transplantation remains the gold standard treatment for end-stage heart failure, offering life-saving intervention to thousands of patients each year. However, the increasing demand for donor hearts and the persistent shortage of available organs result in long waiting lists and significant mortality [1], forcing an allocation process based primarily on clinical evaluations and blood compatibility rather than more time-consuming HLA matching [2]. The traditional preservation method, static cold storage (SCS), has served as the cornerstone of heart transplantation for decades owed to its simplicity, low cost, and proven safety profile for ischemia times under four hours [3]. Despite its indisputable efficacy in this standard setting, its utility becomes constrained when attempting to prolong the preservation window or utilize DCD hearts. These limitations hinder the ability to effectively expand the donor pool beyond current logistical boundaries.

In response, machine perfusion (MP) has emerged as a transformative method of donor heart preservation, encompassing approaches such as normothermic (NMP), hypothermic (HMP), and subnormothermic (SNMP) perfusion. These systems circulate oxygenated, nutrient-rich solutions around the heart [4], often allowing for real-time assessment of graft viability [5]. A critical determinant of these MP techniques is the temperature at which the heart is maintained, as it directly influences myocardial metabolism and post-transplant recovery [6]. Yet, despite promising innovations, the ideal temperature for ex situ preservation is the subject of a debate that has become increasingly dynamic [7]. The central conflict is no longer a simple trade-off between the metabolic monitoring offered by normothermia and the metabolic protection of hypothermic strategies. Recent clinical developments are beginning to blur these lines, testing the core justifications for each approach in real time. This review, therefore, is conducted at a critical juncture where the established value propositions of these technologies are being challenged, making a comprehensive analysis essential to navigate the future of heart preservation.

## 2. Overview of Heart Perfusion and Preservation Techniques

### 2.1. Static Cold Storage

After inspection and irrigation, SCS involves packaging the heart in a sterile bag filled with saline or cold preservation solution and kept at 4 degrees Celsius [2]. The bag is then sealed within two additional sterile layers to prevent contamination and contact-mediated hypothermic injury, which may contribute to graft failure [8]. The sealed heart is then stored in a rigid container filled with a cold solution and placed in an ice-filled cooler [2]. SCS minimizes oxygen and energy requirements of the heart by preventing contraction and reducing temperature [9], thereby delaying ischemic injury and making it a practical and cost-effective method widely adopted since the inception of heart transplantation. SCS remains the undisputed standard for preservation times under four hours, consistently demonstrating adequate protection and excellent outcomes for standard-criteria donor hearts regardless of recipient risk category [10].

However, SCS has notable limitations outside of this safe window. For durations exceeding four hours, the risk of primary graft dysfunction increases significantly due to the extended periods of ischemia and potential temperature fluctuations, which can exacerbate IRI [8]. In addition, a notable exception to the efficacy of SCS has traditionally been DCD hearts, which typically require immediate metabolic support [11,12]. Crucially, recent evidence challenges this dogma; a newly reported method utilizing a controlled, extended, ultra-oxygenated flush followed by SCS has demonstrated success in clinical DCD transplantation, potentially obviating the need for complex perfusion platforms [13]. Still, these limitations have spurred the development of novel preservation techniques that aim to extend preservation times and mitigate ischemic injury.

### 2.2. Temperature-Controlled Hypothermic Static Storage System

Due to the limitations of SCS, new advances in cold storage have been developed, such as the Paragonix SherpaPak^®^ Cardiac Transport System (SCTS) (Paragonix Technologies, Inc., Waltham, MA, USA). This single-use, disposable, and non-perfusing storage system can maintain the donor heart in the theoretical optimal preservation range of 4–8 degrees Celsius [14]. By eliminating the temperature fluctuations and uneven cooling present in SCS, outcomes of clinical trials have highlighted a significant decrease in PGD rates and improved survival [14,15]. Despite its advantages over SCS, it is not as simple and inexpensive, but more importantly, it is still only approved for organ preservation for up to 4 h [16]. The reason for the limited preservation window is assumed to be the cold ischemia time without the provision of oxygen and nutrients for the ongoing metabolic activity of the heart. The development of ex situ perfusion devices aims to mitigate this issue and thus provide a better alternative for preservation.

### 2.3. Normothermic Oxygenated Perfusion

The concept of preserving the heart in a normothermic, beating state was first investigated in the 1980s when Hardesty and Griffith demonstrated laboratory success with this novel technique in animal models [17]. They later reported successful outcomes in clinical testing, further supporting the potential of this technique [18]. In contemporary times, the first and only licensed commercially available device utilizing this approach is The TransMedics Organ Care System™ (OCS) (TransMedics, Inc., Andover, MA, USA). This preservation system uses donor blood to perfuse the donor heart, preserving it at a normothermic (34 °C) sinus rhythm state while maintaining its metabolic processes [19]. Simultaneously, it enables the collection of blood gas and lactate samples to monitor function during transport.

This method of preservation theoretically prevents the rapid temperature changes observed in other methods, therefore reducing the risk of IRI. In addition, the combination of nutrient provision and the ability to monitor the heart in the working mode [20] may facilitate longer preservation times, more distant heart procurement, and improved outcomes. A key caveat, however, is that a beating heart does not inherently equate to a functionally competent heart. As assessment often occurs while the heart is unloaded (lacking physiological preload and afterload), it cannot generate true cardiac output or blood pressure, limiting the reliability of predicting post-transplant hemodynamic performance. This underscores the ongoing search for more robust viability markers. Moreover, the technique faces challenges such as high costs and operational complexity, which may hinder its broader implementation.

### 2.4. Hypothermic Oxygenated Perfusion

The first research on ex situ machine perfusion focused on HMP methods [21]. Following extensive preclinical success [22,23], Wicomb et al. conducted the first series of clinical heterotopic heart transplantations using a portable HMP machine, which yielded modest results [24]. These outcomes, coupled with the inability of functional assessment during preservation, led to the discontinuation of HMP methods until recently, when the growing demand to expand the donor pool renewed interest in their development. The first-in-human study of HMP devices for cardiac preservation demonstrated safety and efficacy, encouraging further clinical investigations [25]. More recently, drawing from recent experiences in lung transplantation, supportive preclinical data, and registry data highlighting the benefits of warmer temperatures, HMP at an internal temperature of 10 °C has also been proposed [26].

Although no HMP device has yet achieved commercial availability, the XVIVO Heart Assist Transport™ (XHAT) system (XVIVO Perfusion AB, Gothenburg, Sweden) is the most advanced device in development, currently in the PRESERVE multicenter clinical trial in the United States [27]. Instead of using warm donor blood for perfusion, this portable system perfuses the heart with a cold (8 °C), nutrient- and hormone-enriched cardioplegic solution containing red blood cells [25]. By preserving the heart in the non-beating state, this approach offers advantages such as reduced metabolic demand and mitigation of IRI—a rationale borrowed from abdominal organ preservation where hypothermic oxygenation has been shown to maintain aerobic metabolism and replenish cellular energy stores during preservation [28,29]. Nevertheless, limitations include the inability to provide real-time functional assessment and, as with NMP, the logistical challenges of scaling the technology for widespread use.

### 2.5. Subnormothermic Oxygenated Perfusion

**Figure 1:**
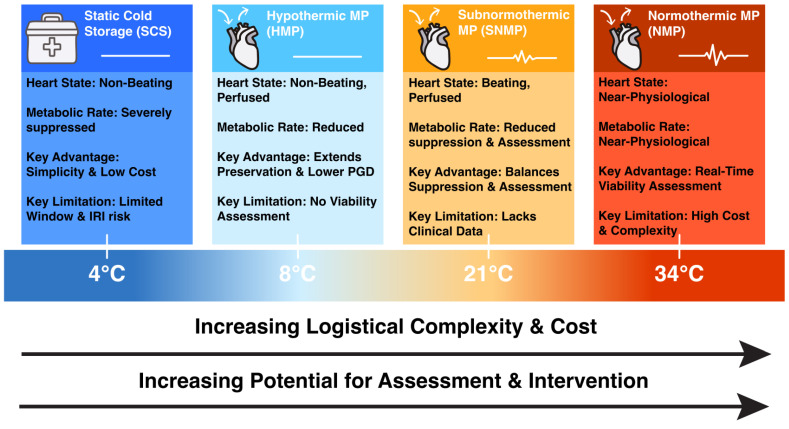
A Comparative framework of myocardial preservation strategies in heart transplantation.

Considering the advantages and drawbacks of both previously mentioned preservation techniques, SNMP has emerged as an innovative strategy. This method bridges the gap between normothermic and hypothermic techniques and acts as a middle ground, preserving the heart at room temperature (21 °C) [30]. Facilitated by convincing preclinical studies [30,31,32], SNMP is believed to combine the benefits of reduced metabolic demand observed in hypothermia with improved oxygenation and nutrient delivery closer to physiological conditions. The moderate temperature could avoid extreme suppression and temperature changes associated with hypothermic methods, while also allowing for functional assessment and graft optimization prior to transplantation, as seen in NMP. However, due to its novelty, further research is mandated to establish standardized protocols, particularly regarding the optimal temperature range and composition of perfusates [33]. These distinct temperature-based strategies are summarized in Figure 1.

## 3. Comparative Analysis of Traditional and Novel Methods

### 3.1. Preserving the Myocardium

When comparing preservation methods, it is vital to consider the preservation efficacy of each technique. This would entail assessing the way each method maintains the structural and functional integrity of the heart, and comparing each one to the current clinical standard, ice-cold storage.

To begin with, the hypothermia induced by SCS does not completely halt metabolism but significantly decelerates biochemical reactions, in doing so decreasing the degradation of essential cellular components necessary for organ viability [34]. Hypothermia also delays the lysis of organelles such as lysosomes, which would otherwise release autolytic enzymes that cause apoptosis. However, theoretical concerns exist regarding storage at extreme temperatures (below 0 °C), which can lead to rapid ATP depletion, impaired endothelial-dependent relaxation, and IRI [35,36,37]. While SCS also possesses the risk of inadvertent uneven cooling if packaging protocols are not strictly followed, this does not reflect the method’s efficacy when performed correctly [8]. The debate surrounding ‘freezing injury’ was historically ignited by Hendry et al., who suggested that donor hearts might be exposed to sub-zero temperatures during transport [38]. However, this assertion has been effectively refuted by subsequent investigations, and more recent analyses indicate that such injury is not inherent to the method when standard protocols are observed. Thus, the risk of cold injury in SCS appears to be overstated in earlier literature [39,40].

Another underappreciated factor is contact-mediated hypothermic injury, which occurs during transport with SCS and has been identified as a contributing cause of graft failure [8]. Unlike SCS, machine perfusion (MP) techniques eliminate these preservation challenges by maintaining the heart at a consistent, controlled temperature range—higher than that used in SCS—while also preventing direct contact with excessively cold or normothermic surfaces that could trigger rapid temperature shifts and/or graft injury [19,25,30].

To counteract the inevitable intracellular acidosis and energy stress of prolonged SCS [11], MP at the various temperatures preserves the donor heart by supplying oxygen and the necessary nutrients to facilitate continuous aerobic metabolism, preventing the production of acidic metabolites and maintaining a more stable and viable tissue pH [30,41,42,43,44]. To further support this, MP has the ability to prevent the accumulation of toxic metabolites from anaerobic processes, such as lactate and adenosine [41], by continuously flushing out waste products during preservation. The prolonged build-up of these toxic metabolites is thought to lead to poor ventricular function or facilitate the generation of free radicals upon reperfusion of the organ [41]. All temperatures of MP can implement this function in preservation, as evidenced in the most established NMP [42] and HMP [43] systems, thereby improving preservation efficacy and graft viability.

An advantage of NMP is its ability to preserve the function of myocardial ion pumps at physiological temperatures, which aids in preventing ion imbalances and maintaining cell homeostasis and pH [7]. Hypothermic temperatures lower the activity of these ion pumps responsible for the recovery to baseline levels, potentially increasing the risk for injury upon reperfusion [45]. To contradict this, HMP has proved its capacity to improve metabolic preservation from the current standards by maintaining a higher rate of functional recovery in heart grafts [43]. Research has suggested that preservation through HMP enhances post-transplant ventricular contractility, cardiac output, and energy stores, while reducing oxidative injury and apoptosis [43,46,47,48]. On another note, subnormothermic temperatures in early studies have conveyed evidence of safeguarding myocyte and endothelial integrity in experimental DBD and DCD heart models compared to SCS [49,50]. SNMP is hypothesized to combine the advantages of maintaining homeostasis at near-physiological conditions with the cytoprotective nature of a semi-hypothermic state [30,31].

Despite the substantial preservation efficacy and success of each system, certain data remain concerning and warrant consideration. For instance, multiple studies on the OCS have reported a notable number of preserved hearts being discarded for various reasons. Some donor hearts displayed suboptimal function or failed to meet transplantation criteria [51], while in other cases, preservation was unsuccessful due to machine malfunctions in the NMP system during transport [52,53]. This exhibits a fundamental difference in the risk profile of the two technologies, as a disruption in function or power through NMP exposes the heart to detrimental warm ischemia time (WIT), whereas HMP system failures revert to standard SCS at a less harmful temperature [54].

Conversely, an alarming quality of hypothermic systems is the formation of edema during preservation and upon reperfusion [11,23,47]. The onset of edema could potentially compromise myocardial perfusion by elevating vascular resistance, which can result in varying levels of oxygenation for cardiomyocytes [55]. Additionally, edema has been linked to a decline in diastolic function [56]. Interestingly, the cardiac edema that arises in HMP studies seems to be easily reversible after transplantation and has not been shown to negatively impact cardiac function [7]. Currently, SNMP’s main limitation is the need for further research to evaluate its ability to effectively address the shortcomings of both NMP and HMP as a middle-ground technique. This need is underscored by a study showing that lowering the temperature from normothermic to subnormothermic levels can impair the functional performance of porcine ex vivo kidney perfusion [57].

### 3.2. Impact on Ischemia–Reperfusion Injury and Mitochondria

The sudden reintroduction of oxygen during SCS greatly elevates the risk of IRI, a process that involves complex cascades of immune activation, mitochondrial dysfunction, and oxidative stress [58,59]. Severe IRI leads to irreversible mitochondrial damage, triggering mitophagy and mitochondrial loss. This directly disrupts cellular energy production and can result in apoptosis or necrosis, both of which drastically disrupt graft function [58]. Additionally, mitochondrial damage indirectly promotes the generation of free radicals and oxidative stress, which cause extensive harm to DNA, proteins, other organelles, and the plasma membrane, further contributing to graft dysfunction. Furthermore, IRI has been found to cause endothelial dysfunction, which can lead to chronic allograft vasculopathy [60] and myocardial cell death [61], posing a serious threat to long-term graft viability and acting as a fundamental predictor of primary graft dysfunction and survival [56]. To further address this, innovative surgical techniques are emerging, such as the ‘beating-heart’ transplantation proposed by the Stanford group, which aims to minimize ischemic insults by avoiding cardioplegic arrest of the donor heart during implantation [62].

Given the detrimental effects of IRI and mitochondrial damage, their prevention during donor heart ex situ perfusion is mandated for an effective preservation strategy. One key advantage of these ex situ systems is their ability to provide continuous oxygenation and minimize ischemic time, the most determinant factor of IRI risk [63]. The continuous oxygenation and the removal of toxic metabolites effectively mimic in vivo conditions and reduce the likelihood of free-radical generation upon reperfusion [41,55]. In addition to these benefits, NMP offers a seamless transition to reperfusion, eliminating large temperature shifts before transplantation. This diminishes the risk of oxidative stress and IRI severity further [44] and maintains a better mitochondrial integrity [64]. However, normothermic conditions increase oxygen demand, and in case metabolic activity is not adequately suppressed, the heart becomes susceptible to prolonged preservation-related stress. Nonetheless, NMP’s capability to examine donor organ functionality prior to transplantation could help address these challenges.

With regard to HMP, studies have demonstrated superior preservation of ATP synthesis, lower endothelin-1 levels, and less apoptosis compared to SCS [64,65], suggesting that hypothermic systems are non-inferior in reducing IRI risk. On the other hand, prolonged exposure to hypothermic conditions has been associated with cold-induced mitochondrial injury and increased susceptibility to IRI [64]. While SNMP is not yet as developed or standardized, it addresses IRI in a similar fashion to NMP by ensuring a relatively smooth temperature transition, maintaining mitochondrial integrity, and preventing excessive energy depletion [24,50,64]. With enough research, SNMP has the potential to offer an optimal balance, combining the advantages of both methods in minimizing IRI risk.

### 3.3. Choice of Perfusate

The selection of perfusate in ex situ heart preservation has been a topic of significant debate due to its substantial impact on preservation efficacy [66,67]. The near-physiological conditions of NMP techniques enable the use of blood-based perfusates, which have been shown to better preserve myocardial function and minimize injury [68]. On the contrary, the reliance on blood-based perfusates introduces significant clinical and logistical challenges. Continuous mechanical pumping poses a risk of red cell lysis, leading to the accumulation of cell-free hemoglobin and inflammatory mediators that may injure the graft. Furthermore, exposure to donor blood carries the risk of recipient allosensitization. From a practical standpoint, non-blood-based (acellular) perfusates offer a distinct advantage by eliminating the complex requirements for blood storage and transport, providing a streamlined, ‘off-the-shelf’ solution that mitigates these immunogenic and mechanical risks [68]. Subnormothermic temperatures have been found to optimize the use of blood-based perfusates more effectively than HMP systems, as the temperature remains high enough to prevent impaired oxygen dissociation and potential coagulation. At the same time, subnormothermic conditions allow for the use of crystalloid perfusates at lower temperatures than NMP, which improves oxygenation since gases are thought to dissolve more readily in colder fluids [42,69]. Nevertheless, a crystalloid perfusate known as Somah solution has demonstrated the most favorable impact on functional recovery under subnormothermic preservation conditions compared to other perfusates and temperature settings [31].

### 3.4. Patient Outcomes (PGD Rate & Survival)

In most clinical trials evaluating the success of preservation systems, the primary endpoints are typically Primary Graft Dysfunction (PGD) rates and survival. According to a JHLT consensus statement, PGD is defined as graft dysfunction occurring within 24 h of cardiac transplant surgery for which a specific cause, such as pulmonary hypertension or hyperacute rejection, cannot be identified [70]. However, this definition is often contested, particularly regarding its sensitivity in distinguishing preservation injury from recipient-related factors [71]. Factors contributing to primary graft failure stem from both pretransplant recipient and donor characteristics, including the etiology of heart failure, coexisting medical conditions, and the need for mechanical circulatory support before transplantation [72]. Additionally, with SCS, exceeding the ideal preservation timeframe of four hours significantly increases the risk of PGD and delayed graft function, ultimately leading to poorer morbidity and mortality outcomes [73]. Furthermore, the reported incidence of PGD in SCS ranges from 7% to 36% [74], though recent randomized trials suggest a lower estimate, closer to 5%; however, in most of these cases, ischemic time was under four hours [75]. Given these considerations, the need to enhance ex situ heart preservation outcomes via extracorporeal machine perfusion becomes increasingly evident.

Initially, NMP via OCS underwent a prospective, single-arm, non-randomized study called PROTECT [76], which aimed to evaluate the safety and performance of the system for preservation and transportation. The study demonstrated promising results, including a 100% survival rate at 30 days post-transplantation and comparable rates of PGD incidence to SCS, confirming the feasibility of OCS for heart preservation and transport. Subsequently, the PROCEED trial was conducted, which also included patients requiring mechanical circulatory support (MCS) [77]. The 30-day survival rate in this study was 84%, establishing the non-inferiority of OCS compared to SCS, with similar patient and graft survival rates and comparable adverse event profiles. This was followed by the PROCEED II trial, which directly compared the clinical outcomes of OCS and SCS [53]. The findings indicated similar 30-day patient and graft survival rates between the two groups, with 94% in the OCS group and 97% in the SCS group. Although OCS successfully established non-inferiority as its primary endpoint, it has yet to demonstrate any significant advantage in clinical outcomes over SCS for standard criteria donors [7].

However, the primary value of NMP may lie not in outperforming SCS for standard hearts, but in its ability to safely expand the donor pool by assessing and utilizing higher-risk organs. This capability was highlighted in the recent OCS Heart EXPAND Trial, which focused specifically on extended-criteria donor (ECD) hearts [78]. The trial demonstrated a high utilization rate of 86.7% for these marginal organs, which are often discarded. Crucially, this was achieved with excellent post-transplant outcomes, including a 96.6% 30-day survival rate and a low incidence of severe PGD at 6.7%, results that are comparable to those seen with standard-criteria hearts. These findings underscore that NMP’s key contribution is enabling the confident transplantation of ECD hearts that might otherwise be wasted, thereby significantly increasing the number of available organs for patients in need.

The most established HMP system, XHAT, was evaluated in the NIHP19 trial, a multinational, multicentre, randomized controlled study conducted in Europe [79]. The trial reported PGD rates of 11% in the HMP group compared to 28% in the SCS group, representing a 61% reduction in PGD risk. Although 30-day mortality was lower in the HMP group, the difference was not statistically significant. Moreover, HMP reduced the need for post-transplant MCS, which was required in 19% of HMP recipients versus 27% in the SCS group. HMP significantly lowered the incidence of major adverse cardiac transplant events (MACTEs), with rates of 18% in the HMP group compared to 32% in the SCS group, further reinforcing its protective role against early post-transplant complications. Following this, XHAT was also evaluated in the Australian and New Zealand HOPE trial [74], which reported a very low incidence of severe PGD, with only one case (3%) in the long preservation group. Notably, despite extended preservation times of up to nine hours, the 30-day survival rate remained 100%. Both trials demonstrated clinically meaningful benefits, including a reduction in graft dysfunction and IRI risk, as well as improved survival rates compared to SCS.

However, a critical anomaly warrants attention when interpreting these comparative results. The incidence of PGD in the SCS control arms of these major industry-sponsored trials—reported as high as 28% in the NIHP2019 trial—is significantly elevated compared to historical registry data, where PGD rates typically range between 5% and 10% for standard ischemia times. This discrepancy raises concerns regarding the potential overestimation of machine perfusion efficacy when compared against a control group that appears to underperform relative to real-world standards. As highlighted in recent correspondence regarding the GUARDIAN-heart registry [80], caution is advised when interpreting data where the limitations of standard cold storage may be overstated, thereby inflating the relative benefit of novel devices.

Research on SNMP remains in the early experimental stages; however, preclinical findings indicate promising potential for improving patient outcomes. One notable study [31] investigated the efficacy of subnormothermic conditions using Somah Solution in porcine hearts. The results demonstrated enhanced metabolic stability, reduced ischemic stress, and significantly higher ATP retention compared to hypothermic conditions and other storage solutions. These findings suggest that SNMP may help mitigate early graft dysfunction, potentially leading to better survival rates in clinical applications. Further, an SNMP study on neonatal and pediatric piglet hearts [33] showed stable hemodynamic and metabolic parameters during extended preservation times. It also outperformed traditional cold storage with superior myocardial integrity. However, while these findings are promising, these data cannot be directly compared to the PGD incidence and survival reported in the other two methods, illuminating the demand of more clinical trials. (Table 1)

### 3.5. Preservation Window and Time Extension

Extending the preservation window of donor hearts is one of the main aims in the process of expanding the donor pool and reshaping the landscape of heart transplantation from a regional to a macroregional level (e.g., American, Pan-European, and transcontinental). SCS subjects the heart to periods of cold ischemia responsible for a higher probability of PGD [72] and mortality [73], and can also multiply other deleterious effects of other risk factors such as donor age [81] and left ventricular hypertrophy [82]. These circumstances constrain the viable preservation window of SCS to up to 4 to 6 h for standard criteria patients, as longer preservation times put the heart at risk of the above complications [83,84]. It is thought, however, that with the supply of oxygen and nutrients through machine perfusion, the preservation window can be broadened to allow for more complex surgical procedures in transplantations [74] and increase access to distant donor hearts [44].

NMP’s advantage in preservation time is believed to be derived from preserving the heart in a beating state, keeping the physiological metabolic processes ongoing. Preclinical trials on NMP systems have shown great promise, demonstrating the capability to safely store donor hearts while maintaining functional and metabolic parameters for extensive durations of up to 12 h [5,85,86,87]. In recent years, clinical investigations into OCS-based preservation have reported comparable outcomes to SCS, despite longer ex vivo durations [53,76,77]. In particular, one study documented a successful transplantation using OCS with an out-of-body time exceeding 10 h [88]. Given its clear potential to extend preservation times, NMP could enable heart procurement from greater distances, thus increasing the donor pool [89]. However, an association has been recorded between longer preservation times on OCS and PGD [54]. This is thought to stem from NMP system failures, which expose the graft to WIT, causing severe organ damage.

Preservation with HMP offers the key advantage of switching to a mode of SCS in the event of system failure, thereby avoiding the associated risk of WIT in NMP [54]. This potential safeguard portrays a distinct superiority of HMP over other ex situ preservation methods. Experimental studies on HMP have underscored preservation times of up to 9 h [84] and, in some cases, as long as 24 h [90]. The XHAT device has undergone multicentre clinical trials, with estimated preservation times ranging from 6 to nearly 9 h [74]. This extended timeframe allowed for the retrieval of donor hearts from remote geographical locations across Australia. The positive outcomes of HMP are primarily attributed to its superior ability to maintain graft function and recovery at hypothermic temperatures.

SNMP is an organ preservation technique designed to integrate the benefits of existing systems by maintaining the heart beating, but at a reduced workload, potentially ameliorating preservation times. Piglet heart preservation studies have recorded viability for up to 10 h, implying that SNMP could remove geographical and transportation barriers and, in doing so, enhance organ donor sharing [33]. However, it has been argued that further research is necessary, notably studies of longer preservation periods across the three different temperature-based methods, to elucidate whether SNMP possesses a unique edge over current approaches [44].

### 3.6. Functional Assessment During Preservation

Much of the success of normothermic preservation techniques can be attributed to the ability to monitor the graft in a near-physiological state. The potential to assess the heart in a beating mode was a primary driver for the development of NMP [91]. However, it is critical to recognize that a beating state alone is not a definitive proxy for pump function. A heart may maintain a sinus rhythm even in states of cardiogenic shock; therefore, without evaluating the organ under physiological loading conditions (generating cardiac output against resistance), ‘functional assessment’ remains a misnomer. Current NMP protocols primarily serve to exclude non-viable organs via lactate trends and visual inspection, rather than confirming contractile reserve.

The OCS has the potential for structural and perfusion assessment through contrast echocardiogram [20] and coronary angiography [92]. In addition, the OCS can predict the risk of coronary artery disease (CAD) and, consequently, graft viability by assessing the aortic pressure at the aortic root [19]. Another viability marker is serum lactate, which has been shown to be a highly sensitive and specific predictor of early graft failure [93]. In contrast, the reliability of perfusate lactate as a metabolic biomarker has been questioned [12,51,88], emphasizing the need for further research to develop a more sensitive and effective cardiac assessment technique, an area that is already under investigation [94]. Once established, SNMP is expected to examine cardiac viability similarly to NMP, as it maintains the heart in the working mode, albeit at a reduced workload [32,44].

Viability assessment is the main superiority of NMP over HMP, as viability assessment of hearts in the non-working state has been shown to be less reliable [95,96]. Despite this, HMP still provides viability measures via metabolic parameters, including oxygen extraction (OER), coronary vascular resistance (CVR), and myocardial oxygen consumption (MVO2) [91,97,98,99,100]. Nevertheless, only the latter two are associated with myocardial heart function preservation and remain less accurate than the coronary angiogram capabilities provided by NMP. Given the critical role of mitochondria in IRI, as previously discussed, mitochondrial function has been proposed as a potential future method for graft function, although research in this area remains limited [3].

### 3.7. Therapeutic Interventions

The concept of intervening on the donor heart during preservation to achieve a therapeutic effect has gained increasing interest in recent years [101,102]. For instance, studies have demonstrated the feasibility of safely delivering targeted genes via viral vectors specifically to the donor heart using NMP [103]. Also, NMP offers an inherently advantageous platform for therapeutic applications, as the heart is supported at a near-normal metabolic rate [103,104], facilitating better uptake of treatments. However, delivering novel therapies at lower temperatures is not unachievable, as literature suggests that effective interventions remain possible even in hypothermic conditions [105,106,107]. Furthermore, in cardiac xenotransplantation, preserving the donor heart with HMP has been deemed essential for prolonging survival in primate models [108]. This approach was later incorporated into the human xenotransplantation protocol, further reinforcing its clinical relevance [109]. Pre-transplant therapeutic interventions hold significant promise, not only in enhancing transplantation success rates, but also in potentially rendering marginal donor hearts viable, in that way expanding the available donor pool.

### 3.8. Compatibility with Donor Types

Utilizing extended criteria donor hearts, as well as DCD hearts, the use of which has been on the rise in recent years [110], provides an alternative strategy of expanding the donor pool further. Marginal donor hearts are typically considered less suitable for transplantation due to the presence of risk factors such as reduced left ventricular (LV) function, LV hypertrophy, significant heart valve disease, and age exceeding 60 years [111]. Additionally, despite emerging protocols [13], research largely indicates that SCS renders most DCD hearts unsuitable for transplantation [12]. This limitation is largely attributed to the inevitable WIT between the withdrawal of life support and circulatory arrest, after which the already energy-depleted heart struggles to tolerate additional cold ischemia [112]. This growing use of DCD and marginal hearts, particularly with in situ resuscitation via thoracoabdominal normothermic regional perfusion (TA-NRP), represents a fundamental philosophical shift: moving from merely slowing cellular decay to actively assessing and restoring organ function before retrieval.

Several studies have described that the OCS seems to be more prominent in high-risk cases and DCD hearts [19,90,113] owing to its ability to assess graft viability, expedite recovery, and administer therapies during preservation. The value of NMP for extended-criteria DBD hearts has now been firmly established, with pivotal trial evidence demonstrating that this technology enables the successful utilization of a high proportion of these otherwise marginal organs while achieving excellent post-transplant survival [78,114,115,116]. The OCS has also proved able to preserve DCD hearts with 30 min of WIT for up to 4 h [112,117,118]. This body of evidence reinforces the superior performance of NMP compared to other ex situ systems when dealing with marginal and DCD hearts.

Conversely, HMP has yet to inspire confidence among heart transplant teams for the use of extended-criteria or DCD hearts [56], as, like SCS, the cold ischemic period may be detrimental to these hearts [12]. However, a recent report has documented the first 3 successful cases of HMP preservation in DCD hearts, contradicting previous assumptions [119]. This success may be associated with HMP’s ability to preserve energy and support functional recovery of DCD heart grafts, as demonstrated in earlier ex vivo models [6]. Furthermore, HMP was recently successfully used for pediatric DBD and DCD hearts with good initial outcomes. This technology is a significant step forward in increasing heart utilization of small donors [120]. Preclinical SNMP studies have similarly reported robust preservation of structural and functional integrity in DCD hearts, suggesting it could provide an optimal environment for long-term storage [45,121].

While SNMP remains in its early stages, the recent success of HMP with DCD hearts signals a potential paradigm shift. This development directly challenges the core justification for NMP’s high cost and complexity—its heretofore unique ability to assess and utilize these extended-criteria organs. If further studies validate HMP’s efficacy for this donor type, its technically safer and PGD-reducing profile could erode NMP’s primary value proposition and fundamentally alter clinical decision-making for a wider spectrum of donors.

### 3.9. Health Economics and Logistical Considerations

When evaluating the effectiveness of new healthcare technologies, logistical factors such as costs and training requirements are always key considerations [122]. One undeniable advantage of SCS is its negligible cost and straightforward implementation. As ex situ perfusion systems become more broadly available, it is vital to critically assess these practical aspects to justify their integration into clinical practice.

Preservation through MP has been shown to be inherently more expensive compared to SCS, largely due to the specialized equipment and additional resources required [21,123,124]. Contrastingly, some researchers have argued that MP may ultimately reduce overall healthcare costs by shortening ICU stays, decreasing the need for postoperative mechanical support, and minimizing reoperation rates [125]. Consequently, while the initial costs of MP are considerably higher, its long-term cost-effectiveness remains a subject of ongoing debate. This economic argument becomes particularly compelling for HMP, whose demonstrated ability to significantly reduce the incidence of costly PGD becomes a powerful value proposition, especially if its applicability continues to expand to DCD hearts, potentially offsetting its higher initial cost compared to SCS.

Another practical consideration is the staffing and training demands of MP systems, particularly with NMP techniques, which often require larger teams, specialized training, and continuous technical support [42]. Given that subnormothermic methods also maintain the heart in a beating state, it is reasonable to expect similar staffing and training requirements to those of NMP. Hypothermic systems, on the other hand, have been reported to be more straightforward to operate, easier to train for, and less dependent on large medical teams [126]. This practical simplicity may present a notable advantage of hypothermic methods over NMP and SNMP, potentially facilitating smoother integration into clinical practice. Furthermore, these high costs and logistical demands raise significant healthcare equity concerns, as their concentration in well-funded centers risks creating a two-tiered system of care and limiting access to this life-saving technology.

It is also important to acknowledge the emerging role of TA-NRP, which gained traction as a cost-effective alternative to MP. However, despite its lower costs, TA-NRP still requires significant staffing resources and relies on OCS technology for organ transportation [127]. These logistical and economic challenges are driving a visionary rethinking of the entire transplant process. Experts envision a move away from the current ‘hurried logistical organization’ towards a new model involving centralized ‘organ repair centers’ [102]. This model represents the logical endpoint of the technological evolution described, fully realizing the philosophical shift from simply slowing decay to actively restoring organ function prior to implantation [101].

## 4. Identifying Gaps and Future Directions

Despite the tangible advancements in ex situ heart perfusion, there remain significant limitations in the existing literature base and practical barriers that must be addressed before these techniques can become standard of care. First, there is a consistent predominance of non-randomized, retrospective studies in most published data, which diminishes the overall level of evidence quality. Furthermore, the interpretation of comparative efficacy is complicated by inconsistencies in control arm outcomes, where reported complication rates in trials often exceed historical registry benchmarks. Although HMP has outlined encouraging early results, the absolute number of transplantations performed under HMP protocols remains relatively low, and there are even fewer published cases of SNMP. Beyond the paucity of large, multicentre trials, there is a need for direct head-to-head clinical studies to determine whether any single preservation temperature—or method—offers a truly superior clinical advantage over the others.

Moreover, variability in perfusion protocols across centers remains a significant problem. Even when using the same device, centers may differ in target temperature ranges, pressure settings, and perfusion flow rates, as well as in the composition and replenishment intervals of perfusates. Such inconsistencies make it challenging to compare data from different studies, slowing the development of universal guidelines and hampering collaborative research efforts. A related issue is the lack of standardized viability criteria, which exacerbates the heterogeneity in clinical outcomes. Currently, functional assessments such as echocardiographic evaluations or coronary angiography may be used in normothermic or subnormothermic systems, while hypothermic protocols often rely on metabolic markers like coronary vascular resistance or oxygen consumption. Even a seemingly straightforward metric such as perfusate lactate clearance has no universally agreed-upon threshold for acceptable graft function, leading to ambiguity over when a heart can be confidently utilized versus discarded.

In addition, donor heart variability poses a considerable challenge. Most published results for ex situ perfusion systems focus on standard-criteria donor hearts, yet many transplant programs increasingly depend on extended-criteria or DCD donors. Hearts with advanced donor age, preexisting comorbidities, or longer warm ischemic times may respond differently to varying temperature strategies and perfusate compositions. For instance, hearts from DCD donors may be more susceptible to even brief periods of inadequate oxygenation, thereby limiting the margin for error in normothermic or subnormothermic approaches. This donor variability underscores the importance of individualized protocols and large-scale studies capturing a wider spectrum of donor characteristics.

Perfusate selection remains a critical area for optimization. Given the logistical and immunological downsides of blood-based perfusates, future research should prioritize the development of advanced acellular solutions capable of supporting metabolic demands without the risks of hemolysis or sensitization. While standardized solutions exist for cold storage, the advent of hypothermic or subnormothermic machine perfusion has led to new proprietary perfusates, often enriched with oxygen carriers, nutrients, and buffers. The optimal perfusate likely varies by target temperature, organ pathology, and even logistical factors like anticipated transport duration, yet little consensus has emerged on how best to tailor these fluids.

From a practical standpoint, resource constraints continue to impede widespread implementation of ex situ perfusion devices. Specialized equipment can be prohibitively expensive, and the need for highly skilled technicians or perfusionists adds another layer of cost and logistic complexity. This challenge is magnified in regions with limited healthcare budgets, where equity in accessing these advanced technologies becomes questionable. Additionally, the risk of device malfunction over longer preservation times cannot be overlooked. Any unexpected breakdown in a normothermic or subnormothermic system can translate to catastrophic warm ischemia, unless robust backup protocols (e.g., prompt transition to static cold storage) are in place.

Significant global disparities in legal and ethical frameworks continue to fragment clinical practice, further complicating the adoption of advanced strategies. For instance, while TA-NRP has seen rapid adoption in countries like Spain [128], the United Kingdom imposed a moratorium on TA-NRP in 2019 until further evidence is available to ensure the technique complies with national legislation [129]. Similarly, the technique faces resistance in the United States due to differing interpretations of the ‘Dead Donor Rule’ [130]. Furthermore, the mandatory ‘stand-off’ period varies widely, ranging from 2 to 5 min in most regions [2] to 20 min in Italy [131]. These legislative inconsistencies directly impact warm ischemia times and organ viability, creating a ‘regulatory lottery’ that complicates the standardization of DCD protocols. Compounding these systemic hurdles is the inherent difficulty of generating evidence for smaller populations; pediatric and high-risk groups remain significantly understudied in ex situ perfusion trials due to low transplant volumes, which complicates efforts to gather the statistically meaningful data necessary to refine protocols for these vulnerable patients.

At last, while short-term endpoints—like PGD rates or immediate survival—are becoming better characterized, long-term outcomes remain a notable blind spot. Large-scale registries and extended follow-up studies tracking chronic rejection, allograft vasculopathy, and late survival are crucial to confirm whether the metabolic and functional benefits of ex situ perfusion translate into robust longevity gains. Recent experimental data also hint at hybrid or cyclical approaches, such as alternating periods of hypothermia and normothermia, as potential strategies to further optimize myocardial protection [132]. However, these innovative protocols remain at an exploratory stage and necessitate rigorous validation in both animal models and human clinical trials.

To bridge these gaps, we envision a fundamental shift in philosophy: moving beyond simple device comparisons toward a risk-stratified ecosystem. A ‘one-size-fits-all’ approach is neither economically sustainable nor clinically necessary. We propose that standardization should be built upon two central pillars. First, for the ‘simplest scenarios’—such as short-distance, standard-criteria DBD hearts—optimized static cold storage remains the most efficient strategy. However, this requires replacing the variability of ice coolers with validated, temperature-controlled devices (e.g., maintaining 4–8 °C) [14] to eliminate freezing injury and ensure consistent protection. Second, targeted machine perfusion should be reserved specifically for elevated-risk scenarios, such as DCD donors or extended-criteria grafts. In this context, the machine serves not merely as a transport device, but as a dynamic ‘bioreactor’, providing a platform for diagnostics, therapeutic intervention, and personalized organ optimization prior to implantation. By standardizing the handling of low-risk allografts rather than abandoning cold storage entirely, the field can direct advanced resources where they yield the highest marginal benefit.

## 5. Conclusions

Ex situ heart perfusion has moved beyond a promising innovation to a clinically validated reality that is fundamentally reshaping the landscape of heart transplantation. The central debate is no longer a simple comparison with SCS but a dynamic re-evaluation of the competing value propositions between normothermic and hypothermic strategies, driven by recent, high-impact clinical trial data.

Rigorous randomized trials have now established a strong evidence base for both NMP and HMP. Normothermic perfusion has proven indispensable for expanding the donor pool by enabling the assessment and successful utilization of ECD hearts. Concurrently, hypothermic perfusion has demonstrated promising potential to mitigate the incidence of PGD, particularly in elevated-risk contexts, although the true magnitude of this benefit relative to optimized cold storage remains a subject of ongoing refinement. This evidence signals a paradigm shift. HMP’s demonstrated clinical benefits and safer failure profile, coupled with emerging success in preserving DCD hearts, directly challenge NMP’s established role as the default strategy for higher-risk organs. The decision-making calculus is shifting from a focus solely on functional assessment towards a balanced consideration of metabolic protection, risk mitigation, and economic viability. We advocate for a standardized, risk-stratified model built on two pillars: optimized static cold storage for standard-risk donors and targeted machine perfusion for high-risk or extended-criteria grafts.

Moving forward, the critical knowledge gap is no longer whether MP is superior to cold storage, but how to optimally deploy it. Priority should be given to direct, head-to-head randomized trials comparing NMP and HMP across different donor types, particularly for DCD and extended-criteria hearts. Furthermore, SNMP remains a critical area for investigation; while still in early experimental stages, its potential to combine the benefits of both approaches, particularly for niche applications like pediatric hearts, warrants dedicated research to translate its preclinical promise into clinical reality. The ultimate goal is to develop evidence-based, donor-specific protocols, transforming ex situ perfusion from a transport tool into a therapeutic ‘bioreactor’ that maximizes the potential of every donated heart.

## Figures and Tables

**Table 1:** Key Clinical and Preclinical Studies of NMP, HMP, and SNMP Systems [31,33,53,74,76,77,78,79].

Preservation Method	Study Name	Sample Size	Mean Preservation Time	PGD Incidence (MP vs. SCS)	Survival (30-Day)	Primary Endpoint	Key Finding/Clinical Implication
**Normothermic Machine Perfusion (NMP)**	PROTECT I (2007) [76]	20 transplanted (human)	222 ± 54 min	Not compared	100%	Safety & Performance	Established initial safety and feasibility of OCS for transport.
	PROCEED I (2008) [77]	13 transplanted (human)	Not reported	15.4%	84.6%	Safety & Effectiveness	Established safety, including in higher-risk MCS patients.
	PROCEED II (2015) [53]	N = 130 (NMP: 62/67; SCS: 63) (human)	211 min	12% vs. 14%	94% vs. 97%	30-day survival	Established non-inferiority of OCS to SCS for standard-criteria donors.
	OCS Heart EXPAND (2024) [78]	150 transplanted (human)	Not reported	6.7% (severe PGD)	96.6%	Composite of 30-day survival & absence of severe PGD	High utilization (86.7%) of extended-criteria donors with excellent survival, confirming NMP’s value in expanding the donor pool.
**Hypothermic Machine Perfusion (HMP)**	NIHP2019 trial (2024) [79]	N = 203 (HMP: 100; SCS: 103) (human)	Not yet published	11% vs. 28%	No significant difference	Composite of major adverse cardiac transplant events	HMP significantly reduced PGD by 61% and major adverse cardiac events.
	Australian and New Zealand Trial (2024) [74]	N = 44 (HMP: 22; SCS: 22) (human)	414 min	5% vs. 19%	100%	Severe PGD incidence	HMP safely extended preservation times to ~7 h and significantly reduced severe PGD.
**Subnormothermic Machine Perfusion (SNMP)**	Somah Solution Preclinical Study (2014) [31]	Porcine model	5 h	Not applicable	Not applicable	Metabolic stability	Demonstrated superior ATP retention and reduced ischemic markers vs. SCS in an animal model.
	SNOP for Neonatal and Pediatric Hearts (2023) [33]	Piglet model	10 h	Not applicable	Not applicable	Hemodynamic stability	Showed the feasibility of extended (10-h) preservation for pediatric-sized hearts.

## Data Availability

No new data were created or analyzed in this study. Data sharing is not applicable to this article.

## Abbreviations

The following abbreviations are used in this manuscript:

SCS	Static cold storage
MP	Machine perfusion
NMP	Normothermic machine perfusion
HMP	Hypothermic machine perfusion
SNMP	Subnormothermic machine perfusion
IRI	Ischemia–reperfusion Injury
SCTS	SherpaPak^®^ Cardiac Transportation System
OCS	Organ Care System™
XHAT	XVIVO Heart Assist Transport™
WIT	Warm ischemia time
PGD	Primary graft dysfunction
JHLT	Journal of Heart and Lung Transplantation
MCS	Mechanical circulatory support
MACTEs	Major adverse cardiac transplant events
ATP	Adenosine triphosphate
DNA	Deoxyribonucleic acid
CAD	Coronary artery disease
DCD	Donation after circulatory death
DBD	Donation after brain death
TA-NRP	Thoracoabdominal normothermic regional perfusion
OER	Oxygen extraction ratio
CVR	Coronary vascular resistance
MVO2	Myocardial oxygen consumption
ECD	Extended-criteria donor
LV	Left ventricular
